# Management of patients with mesh perforation into viscus following pelvic mesh surgery

**DOI:** 10.1007/s00345-025-05512-9

**Published:** 2025-02-28

**Authors:** Yu Hwee Tan, Krishanthy Thayalan, Hannah Krause, Vivien Wong, Judith Goh

**Affiliations:** 1 Queensland Health, Gold Coast, QLD Australia; 2 University of Queensland, Brisbane, QLD Australia; 3 Griffith University School of Medicine, Gold Coast, QLD Australia; 4 Greenslopes Private Hospital, Brisbane, QLD Australia; 5 Department of Urogynaecology, Varsity Lakes Day Hospital, 2 Lake Street, Varsity Lakes, QLD 4227 Australia

**Keywords:** Mesh erosion, Mesh perforation, Mesh in viscus, Mesh complications, Management, Pelvic mesh

## Abstract

**Purpose:**

Pelvic mesh has been used for the treatment of pelvic organ prolapse (POP) and stress urinary incontinence (SUI). Between 3 and 20% suffer complications with debilitating long-term outcomes. Uncommon complications include mesh perforation into viscus however there is minimal published data regarding outcomes following surgical management.

**Methods:**

A retrospective observational study of patients with diagnosis of mesh in viscus at three tertiary urogynaecology units was performed to report on clinical outcomes following surgical management.

**Results:**

Fifty-eight patients were diagnosed with mesh in viscus following cystourethroscopy and thorough examination of vagina/rectum. Mesh involved included mid-urethral slings—retropubic (36.9%), transobturator (18.5%), single incision slings (10.8%); transvaginal POP mesh (15.4%); sacrocolpopexy (13.8%); uncertain type (4.6%). Viscus involved included bladder (39.7%), urethra (50%), bladder and urethra (3.4%), and rectum (6.9%). Main presenting symptoms included mixed urinary incontinence (UI) (75.9%), recurrent urinary tract infections (rUTIs) (48.3%) and rectal/pelvic pain (56.9%). Fifty-one patients underwent mesh excision and viscus repair, with successful repair in all (100%). 48% had complete mesh excision. Mean follow up was 9.5 months (range 0.5–96 months). Post-operatively, there was a statistically significant reduction in rUTIs (p = 0.0004) as well as pain (p = 0.000005). None had recurrent mesh erosion, lower genitourinary tract fistula or wound breakdown.

**Conclusions:**

All patients required thorough examination and cystourethroscopy for diagnosis. Surgical management of mesh in viscus appears to have low morbidity and is shown to reduce symptoms of rUTIs and pain as well as risk of recurrent mesh erosion and fistulae.

## Introduction

Pelvic mesh has been used for management of pelvic organ prolapse (POP) and stress urinary incontinence (SUI). Complications can occur in up to 3% of women who have mid-urethral slings (MUS), up to 4% with sacrocolpopexies; and up to 20% with transvaginal mesh (TVM) for POP. [1] Serious complications include mesh perforation into viscus, resulting in debilitating long-term outcomes [2, 3]. Many patients have significant diagnostic delay, with mean interval 18 to 60 months between MUS placement and recognition of urethral perforation [4–6]. Intra-operative bladder perforation can be up to 4% for MUS but is usually identified with intraoperative cystoscopy and the sling re-positioned. Perforation of surrounding viscus and mesh erosion is poorly reported for TVM as available studies and mesh kits are diverse [7, 8].

Unexpected findings of mesh in viscus are encountered in 3% of patients undergoing surgical treatment of vaginal mesh exposure [9]. This highlights the importance of initial investigations such as detailed vaginal/rectal examination and cystourethroscopy. The AUGS-IUGA Joint Statement on the management of mesh-related complications states that office-based pelvic examinations are first-line diagnostic tools with further investigations dependent on patient specifics, index procedure, and symptoms [10]. Further recommendations include performing cystoscopy, vaginoscopy, digital rectal exam and imaging modalities as appropriate.

The AUGS-IUGA Joint Statement [10] provided a Grade C recommendation that patients with MUS in bladder or urethra can consider endoscopic removal approaches. While endoscopic procedures have lower morbidity, they may have higher recurrence rates [11, 12]. Recurrent mesh perforation into viscus is an indication for complete MUS excision (Grade B recommendation). For management of TVM in viscus, Cundiff et al. [1] provided a Grade B recommendation with limited evidence that perforation involving the lower urinary tract requires more radical surgery. As opposed to MUS, endoscopic treatment of TVM in the urethra/bladder generally is inadequate, resulting in recurrent complications. Cystotomy or urethrotomy with excision and closure may be more effective and may be accomplished vaginally [13].

This paper presents the management and outcomes of patients presenting to three tertiary specialist urogynaecology centres with mesh perforation into viscus. The aim is to report clinical outcomes following surgical management of complications, specifically mesh removal in patients with mesh in viscus, as there is limited published data in this area.

## Materials and methods

A retrospective cohort study was performed of patients who had been diagnosed with pelvic mesh in viscus and had undergone management at three tertiary urogynaecology centres. Ethics committee approval was obtained from Greenslopes Research and Ethics Committee (Protocol 18/62 GPH), Gold Coast Health Human Research Ethics Committee (HREC/2019/QGC/52891) and Metro South Human Research Ethics Committee (HREC/2023/QMS/94812). Patients who had undergone management of mesh in viscus from April 2007 to April 2023 were identified from hospital records. Paper and electronic hospital records were reviewed. Data was collected on patient demographics, mesh type and details of original surgery, presenting symptoms, prior treatments in other centres, investigations, management and surgical/post-operative outcomes and symptoms. All patients who had been diagnosed with mesh erosion into viscus were eligible for inclusion.

All patients underwent standardised history-taking and clinical examination. Medical records of previous mesh implantation surgery were obtained. All patients underwent standardised examination of vagina/rectum and cystourethroscopy for diagnosis, performed under general anaesthesia (GA) or sedation. Cystourethroscopy involved use of a 70-degree, 30-degree and/or a 0-degree cystoscope. The bladder was distended to maximum capacity, to exclude mesh perforation not apparent at lower volumes and other pathology.

If there was suspicion of genitourinary fistula, a methylene-blue dye test was performed. This involved instilling at least 200 ml dilute methylene-blue dye into the bladder via Foley catheter, while observing for vaginal leak with aid of a speculum. If there was possible rectovaginal fistula, betadine solution was instilled per rectum via Toomey syringe to assess for vaginal leak.

All patients were counselled regarding their options including conservative or minimally invasive management, partial excision of mesh involved and viscus repair, and complete excision of the mesh involved and viscus repair.

Partial excision of slings or TVM involved vaginal incisions. Complete excision of implants involved vaginal and groin incisions for trans-obturator slings and TVM; vaginal and small Pfannenstiel incision for retropubic slings and sacrocolpopexy mesh.

### Repair of bladder

Surgical repair of bladder was undertaken transvaginally or transabdominally depending on the location of mesh erosion. Ureteric catheterisation was performed if mesh erosion was close to the ureteric orifices. Following excision of bladder mesh, the extent of the bladder defect was identified, and the bladder dissected from surrounding vagina to allow tension-free repair. The bladder was repaired in two layers with absorbable sutures (2–0 polyglactin). Following this, a methylene-blue dye test was performed to confirm a watertight closure. The Foley catheter was left in situ for two weeks with oral antibiotic cover, followed by an outpatient methylene-blue dye test to exclude fistula and an immediate trial of void (TOV) if negative.

### Repair of urethra

Surgical repair of the urethra was carried out transvaginally following transvaginal excision of urethral mesh erosion. Excision of urethral mesh was performed through the mesh perforation sites without additional urethrotomy. Dissection of the urethra from surrounding vaginal tissue was performed to allow tension-free repair. Closure of the urethral defect was performed in two layers with interrupted absorbable sutures (3.0 polyglactin), in a horizontal manner to reduce risk of urethral stricture.

A methylene-blue dye test was then performed to ensure watertightness. Following urethral repair, a labial fat flap or modified Martius flap was routinely performed. A Foley catheter was left in the bladder for 3 weeks, followed by an outpatient methylene-blue dye test to exclude fistula and TOV if negative.

With regards to our units’ technique for performing a labial fat flap, it has some similarities to that described by Wilson et al. [15]. The key differences include that our unit makes a smaller incision starting at a level lower than the mons pubis in order to avoid a large labial scar or labial distortion. Generally, suture ligation is not required as the flap is divided with diathermy. Strict haemostasis is observed and a drain is not routinely placed within the labial wound.

Primary outcome was successful viscus repair, as determined by a negative methylene-blue dye test at two to three weeks postoperatively. Secondary outcomes included resolution of presenting symptoms at 3 months including pain, UI and rUTIs. All patients were followed up at 6 weeks and 3 months post-operatively, and longer when clinically required. Data was analysed using Microsoft Excel and Stata version 18 (Stat, Statacorp LLC, Texas, USA). Statistical analysis of pre-operative and postoperative symptoms was performed using McNemar’s chi-square test. A p value < 0.05 was considered statistically significant.

## Results

Fifty-eight patients were diagnosed with mesh erosion into viscus from April 2007 to April 2023 with mean age 65 years (SD 10.28) and mean BMI 28.7 (SD 4.44). Forty-six (79.3%) patients were referred following the Australian Senate Inquiry into transvaginal mesh implants in March 2018 [14]. Twenty-five patients (43.1%) were referred to a specialised state-wide mesh complication management service after establishment of the service in April 2019. Table 1 provides a summary of our patient population.

**Table 1:** Baseline characteristics of patient population

Demographics	Mean (SD)
Age	65y (SD 10.28)
BMI	28.7 (SD 4.4)

Type of pelvic mesh	Number (%)
Retropubic mid-urethral sling	24 (36.9%)
Transobturator mid-urethral sling	12 (18.5%)
Single incision slings	7 (10.8%)
Transvaginal mesh for prolapse	10 (15.4%)
Abdominal sacrocolpopexy mesh	9 (13.8%)
Uncertain type	3 (4.6%)

Original region of mesh insertion	Number (%)
Urban centre	38 (65.5%)
Regional centre	11 (19.0%
Interstate	8 (13.8%)
No record available	1 (1.7%)

Public vs private hospital	Number (%)
Public	26 (44.8%)
Private	28 (48.3%)
No record available	4 (6.9%)

Viscus involved	Number (%)
Bladder	23 (39.7%)
Urethra	29 (50%)
Bladder and urethra	2 (3.4%)
Rectum	4 (6.9%)

Presenting symptoms	Number (%)
Mixed urinary incontinence	44 (75.9%)
Recurrent urinary tract infections	28 (48.3%)
Voiding dysfunction	11 (19%)
Pain	32 (53.4%)
Rectal pain	2 (3.4%)
Discharge from perianal sinus	1 (1.7%)
Faecal incontinence	1 (1.7%)

Treatment details at prior units (n = 22)	
Treatment	Number (%)
Partial excision of mesh in viscus endoscopically	10 (45.5%)
Removal of calculi overlying mesh perforation in viscus	3 (13.6%)
Medical management for recurrent UTIs	4 (18.2)
Urethral dilatation for voiding dysfunction	2 (9.1%)
Exploratory laparotomy (Urology)	1 (4.5%)
Repair of urethrovaginal fistula	1 (4.5%)
Laparoscopic anterior resection, mesh excision in diverticular phlegmon anterior to rectum (Colorectal)	1 (4.5%)

The types of mesh implants involved are detailed in Table 1. Seven patients had multiple mesh types. Retropubic MUS included top-down (12.5%, 3), multifilamentous (12.5%, 3), bottom-top (58.3%, 14) and unknown (16.7%, 4). The sites of mesh perforation are detailed in Table 1. Nine (36%, 9/25) patients with bladder mesh erosion had a recognised cystotomy at time of initial mesh insertion—5 sacrocolpopexies, 3 MUS and 1 anterior TVM. Two of the patients with urethral mesh erosion had a documented viscus injury at time of initial MUS insertion. One patient with bladder mesh erosion had had an unrecognised ureteric injury at time of initial mesh insertion and underwent right ureteric reimplantation two months postoperatively. She then had a psoas hitch nine months post-operatively due to ureteric stenosis, followed by ureteric stenting and subsequent nephrectomy.

Presenting symptoms included mixed UI (75.9%, 44), rUTIs (48.3%, 28), voiding dysfunction (19%, 11), and pelvic pain/dyspareunia (53.4%, 32). Thirty-four (58.6%) patients underwent investigations prior to referral and 38% (22/58) received treatment at a prior unit – of those previously investigated, twenty patients (58.8%) did not have mesh erosion identified. The treatment details of the twenty-two patients who received treatment at a prior unit are summarized in Table 1.

**Fig. 1:**
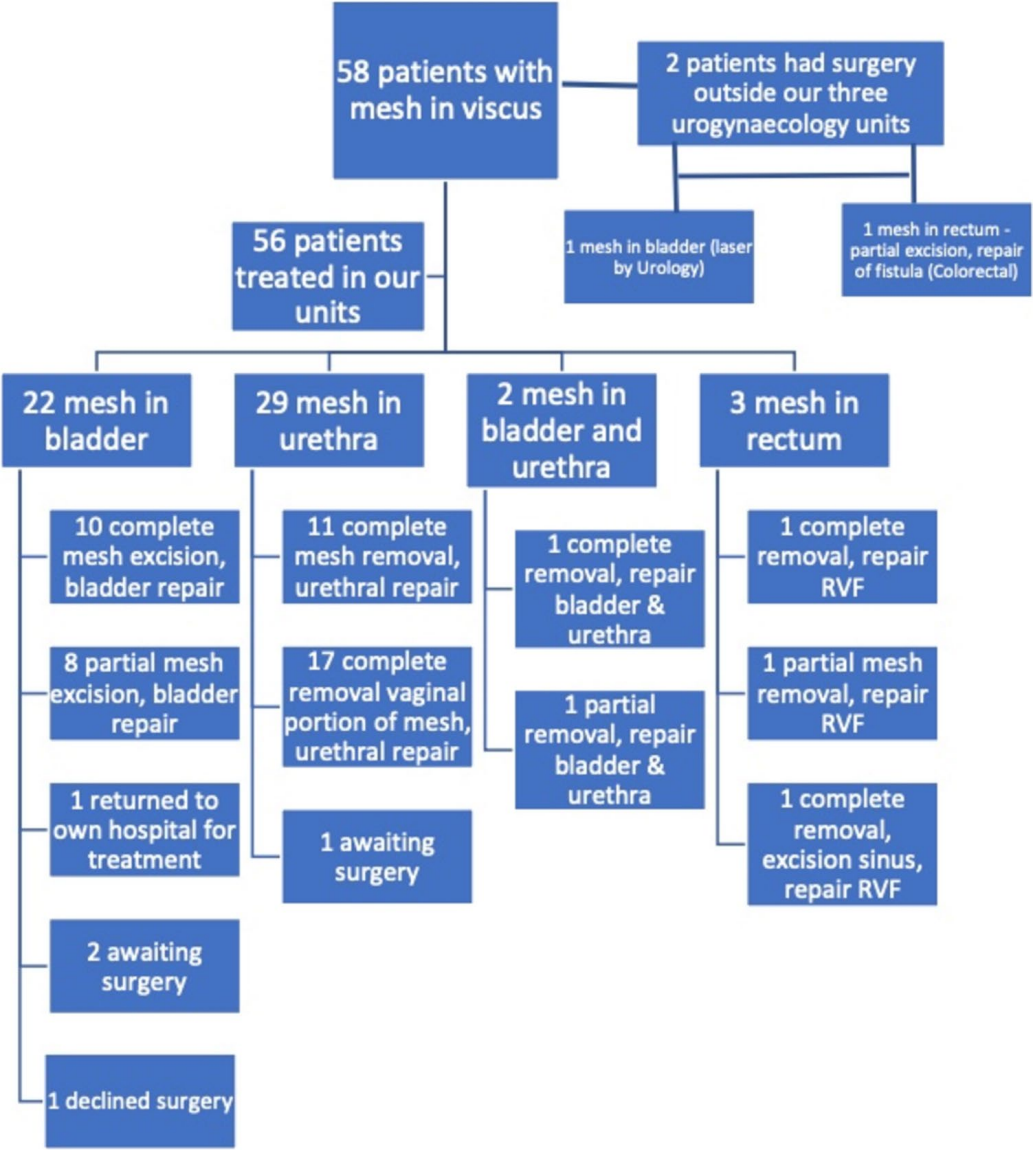
Patient outcomes

Median duration from initial mesh insertion to diagnosis was 8 years (range 1–25 years). Of the fifty-eight patients, fifty-one women had surgery within the three tertiary urogynaecology units for mesh in bladder (n = 18), urethra (n = 28), bladder and urethra (n = 2) and rectum (n = 3). Thirty patients underwent labial fat flap at time of urethral repair. Of the remaining seven patients, one patient underwent laser excision of bladder mesh with the urology team; one patient underwent rectal mesh excision with the colorectal team and the remaining 5 patients declined treatment or are awaiting surgery. The patient who had surgery with the colorectal team did not have recurrent mesh complications. The patient who had laser treatment had recurrent bladder mesh erosion. Figure 1 provides a summary of the patients’ surgical treatments.

The following results relate to the 51 patients treated at our three tertiary urogynaecology centres. All patients with bladder and urethral repair were reviewed at 2–3 weeks postoperatively for methylene-blue dye test and TOV. All patients who had surgery for mesh in viscus were followed up at 6-weeks, 3-months and longer, if clinically indicated. Patients were followed up to an average of 9.5 months (range 0.5–96 months). Repair of viscus injury was successful in all fifty-one patients [100%].

Post-operative complications occurred in 9.8% of patients (5/51). These included medical readmission for atrial fibrillation (n = 1), same-day return to theatre for washout of bladder clots (n = 1), wound infection/collection managed conservatively (n = 2), and pelvic collection requiring CT-guided drainage (n = 1). None required blood transfusions, and all recovered well.

At 3-months follow up, 66.7% of patients had ongoing mixed UI. There was no significant difference in pre-operative and post-operative urinary incontinence symptoms (OR 0.55 95% CI [0.166, 1.609], p 0.225). Among patients with pre-existing pain, 21.2% had persistent pain symptoms. There was a statistically significant reduction in pain post-operatively (OR 0.77 (95% CI [0.009, 0.307], p = 0.000005). There was no de novo cases of post-operative pain. While there was a statistically significant reduction in the symptoms of rUTIs post-operatively (OR 0.15 (95% CI [0.029, 0.506], p = 0.0004), 25% of patients had ongoing symptoms of cystitis. Two patients underwent further mesh removal surgery—one patient, who had a partial mesh removal and urethral repair underwent removal of remaining sling, intravesical Botox, and urethral bulking 12 months later due to continued mixed UI and pain. One patient, who had partial mesh removal and bladder repair, underwent excision of vaginal mesh exposure 28 months later.

## Discussion

Bladder or urethral mesh perforation can present with varied symptoms including pelvic pain, rUTIs, UI, and voiding dysfunction. Patients with rectal mesh perforation may present with rectal pain or abnormal vaginal discharge. Due to the range of presenting symptoms, clinicians should consider excluding mesh perforation into viscus in any patient with previous pelvic mesh surgery.

Although clinical examination is essential and can confirm vaginal mesh exposure, it cannot exclude viscus perforation. Many patients with viscus perforation had concomitant vaginal mesh exposure, mainly anterior. Based on our study, it is essential to perform a thorough vaginal/rectal examination, and careful cystourethroscopy on all patients with pelvic floor symptoms and a history of pelvic mesh implantation, as almost 60% of patients who presented to our units with mesh in viscus had not previously been diagnosed with this. A thorough cystoscopic examination is important to identify mesh in viscus, plan management and avoid complications from unexpected findings. Routine thorough examination and cystourethroscopy allows for extensive patient counselling and informed consent. It also assists with the exclusion of differential diagnosis such as bladder pain syndrome and malignancy.

Since nearly 50% of our patient population was referred to a specialised tertiary state-wide pelvic mesh complication management service following its inception in April 2019, this suggests that specialised services and increased patient awareness can enhance referrals, leading to better detection and management of these significant complications.

Our study demonstrates the effectiveness of surgical treatment for mesh erosion into viscus. All patients treated at the three urogynaecology subspecialty units experienced no recurrence of mesh in the viscus or fistula. Given that there are two experienced fistula surgeons (JG, HK) in these units, mesh removal and reconstruction of the affected viscus were performed or supervised by them. The presence of skilled surgeons to perform or oversee these complex procedures is crucial for ensuring successful surgical outcomes. The combined experience of the two fistula surgeons exceeded 50 years in total with regards to performing fistula surgery. Following urethral repair, we routinely performed a labial fat flap. Given that these patients have high rates of recurrent UI following MUS excision, the majority may require future SUI surgery. Performing a labial fat flap at time of urethral repair allows reinforcement of periurethral fascia and provides tissue between the urethra and vagina, aiming to reduce risk of urethral injury during secondary SUI operations (15).

Surgical management of mesh in viscus can be an effective, definitive, and safe procedure which reduces the risk of recurrent mesh perforation into viscus. Although there was a complication rate of 9.8% in our population, only one required return to theatre.

The rate of recurrent or residual pelvic floor dysfunction, especially UI following sling excision, is high. It is important that in counselling regarding management options, patients are informed regarding the significant risk of recurrent SUI and incomplete resolution of pain and other symptoms. This is important in terms of planning future care and setting patient expectations. Our study has shown that there is a significant reduction in rUTIs and pain following surgical management of mesh in viscus which indicates that this is an effective treatment option.

Limitations of this study include its retrospective cohort design; a prospective study could further reinforce the significance of our findings. Additionally, the absence of validated questionnaire data in reporting outcomes may be a limitation. However, patients were asked about the same preoperative and postoperative symptoms, which provided a clear picture of post-operative outcomes. Our study results may not be broadly applicable, as the patients were treated by highly skilled fistula surgeons. However, these findings highlight the importance for specialised pelvic mesh complication centres to develop expertise in this area of pelvic reconstructive surgery.

A key strength of this study is that it represents, to our knowledge, the largest population of patients who have undergone surgical management of mesh in the viscus. The findings will contribute valuable evidence to the management of these complex complications, an area where optimal management is not yet well understood.

## Data Availability

No datasets were generated or analysed during the current study.
